# Zhuifeng tougu capsules inhibit the TLR4/MyD88/NF-κB signaling pathway and alleviate knee osteoarthritis: *In vitro* and *in vivo* experiments

**DOI:** 10.3389/fphar.2022.951860

**Published:** 2022-09-15

**Authors:** Xiaotong Xu, Naping Li, Yongrong Wu, Ke Yan, Yilin Mi, Nanxing Yi, Xuyi Tan, Gaoyan Kuang, Min Lu

**Affiliations:** ^1^ Department of Orthopedic Surgery, The First Hospital of Hunan University of Chinese Medicine, Changsha, Hunan, China; ^2^ Graduate School, Hunan University of Chinese Medicine, Changsha, Hunan, China; ^3^ School of Traditional Chinese Medicine, Hunan University of Chinese Medicine, Changsha, Hunan, China; ^4^ Department of Orthopedic Surgery, Affiliated Hospital of Hunan Academy of Chinese Medical Science, Changsha, Hunan, China; ^5^ Postdoctoral Research Workstation, Hinye Pharmaceutical Co., Ltd., Changsha, Hunan, China

**Keywords:** Zhuifeng tougu capsules, TLR4, NF-κB, knee osteoarthritis, inflammatory factors, innate immune

## Abstract

**Background:** Knee osteoarthritis (KOA), a chronic degenerative disease, is mainly characterized by destruction of articular cartilage and inflammatory reactions. At present, there is a lack of economical and effective clinical treatment. Zhuifeng Tougu (ZFTG) capsules have been clinically approved for treatment of OA as they relieve joint pain and inflammatory manifestations. However, the mechanism of ZFTG in KOA remains unknown.

**Purpose:** This study aimed to investigate the effect of ZFTG on the TLR4/MyD88/NF-κB signaling pathway and its therapeutic effect on rabbits with KOA.

**Study design:** *In vivo*, we established a rabbit KOA model using the modified Videman method. *In vitro*, we treated chondrocytes with IL-1β to induce a pro-inflammatory phenotype and then intervened with different concentrations of ZFTG. Levels of IL-1β, IL-6, TNF-α, and IFN-γ were assessed with histological observations and ELISA data. The effect of ZFTG on the viability of chondrocytes was detected using a Cell Counting Kit-8 and flow cytometry. The protein and mRNA expressions of TLR2, TLR4, MyD88, and NF-κB were detected using Western blot and RT-qPCR and immunofluorescence observation of NF-κB p65 protein expression, respectively, to investigate the mechanism of ZFTG in inhibiting inflammatory injury of rabbit articular chondrocytes and alleviating cartilage degeneration.

**Results:** The TLR4/MyD88/NF-κB signaling pathway in rabbits with KOA was inhibited, and the levels of IL-1β, IL-6, TNF-α, and IFN-γ in blood and cell were significantly downregulated, consistent with histological results. Both the protein and mRNA expressions of TLR2, TLR4, MyD88, NF-κB, and NF-κB p65 proteins in that nucleus decreased in the ZFTG groups. Moreover, ZFTG promotes the survival of chondrocytes and inhibits the apoptosis of inflammatory chondrocytes.

**Conclusion:** ZFTG alleviates the degeneration of rabbit knee joint cartilage, inhibits the apoptosis of inflammatory chondrocytes, and promotes the survival of chondrocytes. The underlying mechanism may be inhibition of the TLR4/MyD88/NF-kB signaling pathway and secretion of inflammatory factors.

## 1 Introduction

Knee osteoarthritis (KOA) is a chronic osteoarticular disease with a higher incidence during middle age, especially in the joints, and it is associated with the spine, knee, and hip joints. KOA is characterized by degeneration of articular cartilage, subchondral bone sclerosis, and osteophyte formation (Miller et al., 2019). OA is also known as proliferative KOA, hypertrophic arthritis, or senile arthritis. Except for the elderly, the occurrence and development of KOA are closely related to gender (especially in women), obesity, and heredity factors, as well as the individual difference in their response to immunity.

Early joint pain is the first symptom. The degree of pain becomes increasingly serious with increase in joint activity, affecting the quality of life of patients and eventually causing disability (O’Neill and Felson, 2018). Therefore, joint replacement becomes the final choice for patients as it implies a greater surgical risk and economic burden (Hofmann et al., 2011).

Modern medicine recognizes that KOA is a disease of the whole joint, including lesions of various tissues (Malfait, 2016). However, most studies have focused on articular cartilage, which is related to evolution of OA (Wu et al., 2002). However, many research advances exist on the correlation between bone marrow lesions, synovitis, and OA (Pauli et al., 2012; Coughlin and Kennedy, 2016; O’Neill and Felson, 2018). As inflammation is the core of the pathogenesis of OA (Scanzello, 2017), including synovitis, innate immune mechanism, and the inherent inflammation of articular cartilage (Sakalyte et al., 2021), it is the current research hotspot in OA. Furthermore, evidence reveals that the occurrence and development of OA are closely related to an autoimmune mechanism (Barreto et al., 2020).

Unlike the acquired immune system, the innate immune mechanism is mainly involved in tissue repair, wound healing, apoptosis, and cell debris removal, serving as the initial line of defense against infection. The immune cells of the innate immune mechanism mainly include macrophages, granulocytes, mast cells, and dendritic cells. Fast-acting innate immune cells play an important role in inducing inflammation (Tang et al., 2018; McComb et al., 2019). Among them, inflammation (often subclinical) mediated by toll-like receptors (TLRs) and secreted by immune cells leads to osteoclast-like bone erosion (Liu J et al., 2021), thus aggravating the joint inflammatory response.

The TLR family comprises ten types of transmembrane protein receptors in humans (TLR 1–10). TLR 1, 2, 4, 5, 6, and 10 are located on the cell surface, which mostly rely on the myeloid differentiation factor 88 (MyD88) and cytoplasmic adaptor, further activating the inflammatory response with the nuclear factor kappa B (NF-κB). The other portion of TLRs, including TLR 3, 7, 8, and 9, is located intracellularly and can directly activate IRFs across MyD88 to induce inflammatory responses. Within this, the TLR4 receptor is the only two-way TLR that triggers internalization into the intracellular-activated IRF pathway after activation of MyD88. TLR10, the newly discovered TLR in humans, is also the only known anti-inflammatory molecule in the TLR family (Oosting et al., 2014). Its mechanism may be related to inhibition of the NF-κB signaling pathway (Tang et al., 2018; Vrgoc et al., 2018; Fore et al., 2021). Evidence suggests that the secreted inflammatory factors {interleukin -1(IL-1β), tumor necrosis factor-alpha (TNF-α), and IL-6} in the cartilage of patients with OA are elevated, which is closely linked to TLRs (Nie et al., 2019).

The mechanism to slow down the occurrence and development of OA is the current research focus. As a national treasure of the Chinese nation, traditional Chinese medicine (TCM) plays an irreplaceable role in maintaining human health. Zhuifeng Tougu (ZFTG) capsules, a clinical drug approved for OA, are composed of classic famous prescriptions, including Xiaohuoluo pills and the Linggui Zhugan and Jiuwei Qianghuo decoctions, which effectively relieve joint pain and inflammation in patients with OA. It can expel wind, eliminate dampness, dredge meridians and collaterals, dispel cold, and relieve pain. The composition and dosage in the automatic production line of ZFTG are illustrated in Table 1. However, the specific efficacy mechanism for alleviating OA remains unclear. Therefore, this study aimed to investigate the efficacy of ZFTG on rabbit KOA and its effect on TLRs.

**TABLE 1:** Herbs in ZFTG.

Scientific name of the herb	Part used	Latin name of the medicinal material	Family name	Chinese name	Dosage (kg)^a^
*Angelica dahurica* (Fisch.ex Hoffm.) Benth. et Hook.f	Root	*Radix Angelicae Dahuricae*	Umbelliferae	Bai Zhi	48.1
*Atractylodes macrocephala* Koidz	Root and rhizome	*Rhizoma Atractylodis Macrocephalae*	Compositae	Bai Zhu	24.0
*Aconitum kusnezoffii* Reichb	Root	*Radix aconiti agrestis*	Ranunculaceae	Cao Wu	48.1
*Aconitum carmichaeli* Debx	Root	*Radix Aconiti Preparata*	Ranunculaceae	Chuan Wu	48.1
*Ligusticum chuanxiong* Hort	Root	*Rhizoma Chuanxiong*	Umbelliferae	Chuan Xiong	48.1
*Paeonia veitchii* Lynch	Root	*Radix Paeoniae Rubra*	Ranunculaceae	Chi Shao	48.1
*Vigna umbeuata* (Thunb.) Ohwi et Ohashi	Seed	*Semen Phaseoli*	Leguminosae	Chi Xiao Dou	48.1
*Angelica sinensis* (Oliv.) Diele	Root	*Radix Angelicae Sinensis*	Umbelliferae	Dang Gui	24.0
*Pheretima aspergillum* (E. Perrier)	Pheretima removes all the viscera	*Earthworm*	Megascolecidae	Di Long	48.1
*Poria cocos* (schw) Wolf	Sclerotia	*Poria*	Polyporaceae	Fu Ling	96.2
*Saposhnikovia divaricata* (Turcz.) Schischk	Root	*Radix Saposhnikoviae*	Umbelliferae	Fang Feng	24.0
*Glycyrrhiza uralensis* Fisch	Root and rhizome	*Radix Glycyrrhizae*	Leguminosae	Gan Cao	48.1
*Nardostachys jatamansi* (D. Don) DC.	Root and rhizome	*Nardostachyos Root and Rhizome*	Valerianaceae	Gan Song	24.0
*Cinnamomum cassia* Presl	Cassia twig	*Ramulus Cinnamomi*	Camphor	Gui Zhi	24.0
*Ephedra sinica* Stapf	Herbaceous stem	*Herba Ephedrae*	Ephedraceae	Ma Huang	48.1
*Commiphora myrrha* Engl	Resin	*Myrrh*	Burseraceae	Mo Yao	9.6
*Notopterygium incisum* Ting. ex H. T. Chang	Root and rhizome	*Rhizoma seu Radix Notopterygii*	Umbelliferae	Qiang Huo	48.1
*Gentiana macrophylla* Pall	Root	*Radix Gentianae Macrophyllae*	Gentianaceae	Qin Jiao	24.0
*Boswellia carterii* Birdw	Resin	*Frankincense*	Burseraceae	Ru Xiang	24.0
*Gastrodia elata* BL.	Tuber	*Rhizoma Gastrodiae*	Orchidaceae	Tian Ma	24.0
*Arisaema heterophyllum* Blume	Tuber	*Rhizoma Arisaematis*	Araceae	Tian Nan Xing	48.1
*Asarum sieboldii* Miq	Root and rhizome	*Herba Asari*	Aristolochiaceae	Xi Xin	48.1
*Cyperus rotundus* L	Root and rhizome	*Rhizoma Cyperi*	Cyperaceae	Xiang Fu	48.1

The plant name has been checked with (http://www.theplantlist.org).

a Dry weight of the medicinal material.

## 2 Materials and methods

### 2.1 Materials

**FIGURE 1:**
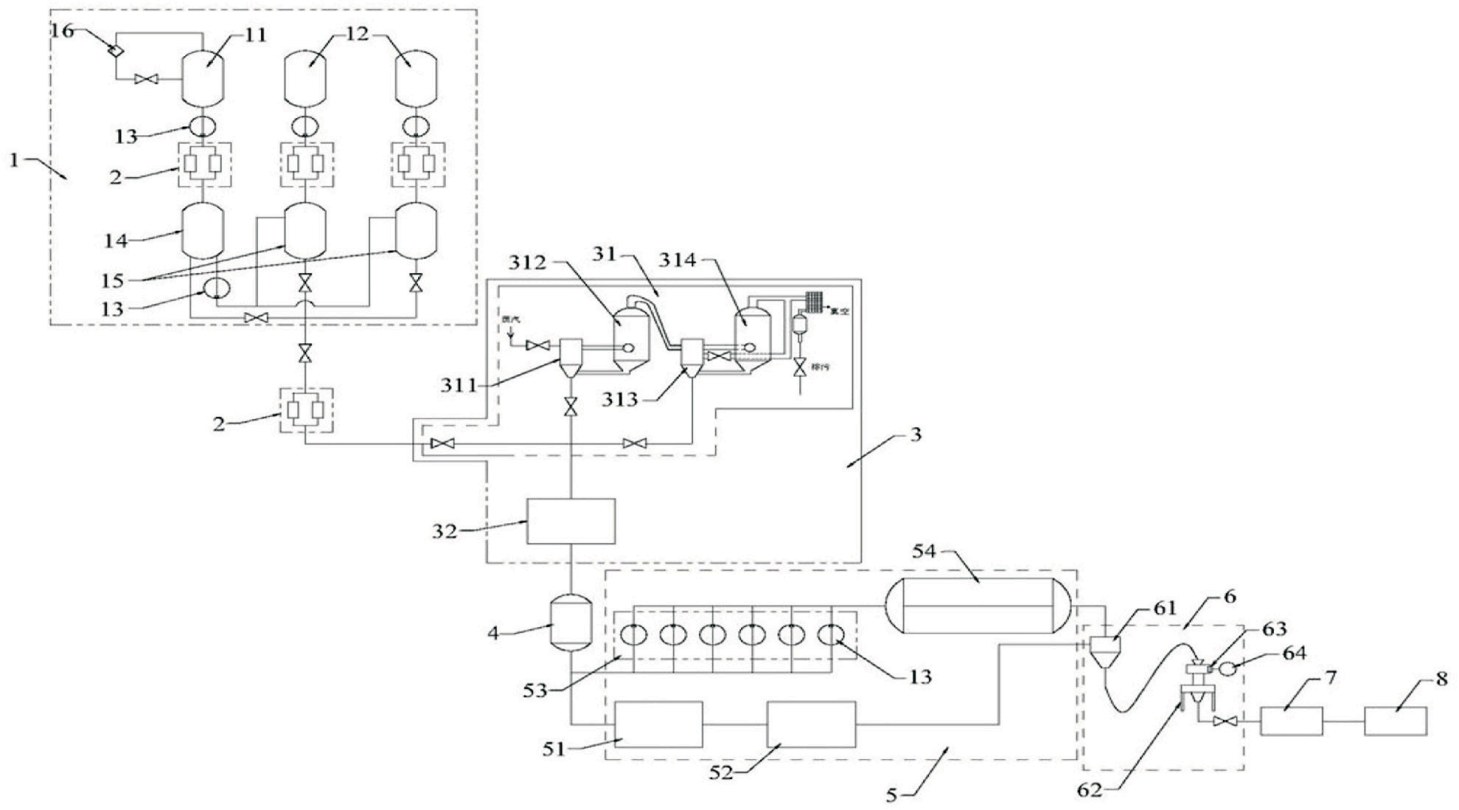
Structure layout diagram of the automatic production line of ZFTG. Description of reference numeral: 1. a liquid medicine extracting device; 2. double filter; 3. concentration device; 4. ingredient tank; 5. drying device; 6. a crushing device; 7. mixing device; 8. automatic packaging device; 11. a first extraction tank; 12. a second extraction tank; 13. transfer pump; 14. a first liquid storage tank; 15. a second liquid storage tank; 16. oil-water separator; 31. double-effect concentrator; 32. spherical scraper concentrator; 311. a single-effect heating chamber; 312. a primary-effect evaporation chamber; 313. two-effect heating chamber; 314. two-effect evaporation chamber; 51. trough mixer; 52. vacuum drying oven; 53. coating device; 54. vacuum belt drier; 61. mixing hopper; 62. crusher; 63. filtration device; 64. vacuum pump. Patent application number: CN 215133530 U.

ZFTG was produced and provided by Hinye Pharmaceutical Co., Ltd. (Batch No. 200505, Changsha, China) and produced according to the automatic production line displayed in Figure 1. The samples were subjected to pretreatment using a combination of liquid–liquid and solid-phase extraction to ensure the stability of drug quality (see Patent CN 11165036A for details). Fingerprint analysis and ultra-performance liquid chromatography (UPLC) demonstrated the effective components of ZFTG. Glucosamine sulfate capsules (GS) were provided by Zhejiang Haizheng Pharmaceutical Co., Ltd. (Batch No. 71812133, Changsha, China). The primary antibodies of TLR2 (66645-1-Ig), TLR4 (19811-1-AP), MyD88 (23230-1-AP), NF-κB (10745-1-AP), and β-actin (60008-1-Ig) were all purchased from ProteinTech Group Inc. (Chicago, IL, United States). NF-κB p65 (ab16502) was purchased from Abcam (Cambridge, UK). IL-6 (bs-6312R), IL-1β (bs-0812R), IFN-γ (bs-0480r), and TNF-α (bsm-33207m) antibodies were purchased from Beijing Bioss Co., Ltd. (China). The primers for all genes were synthesized by Sangon Biological Engineering (Shanghai, China). The primer list is displayed in Table 2.

**TABLE 2:** Sequences of primers used in the qRT-PCR.

Gene	Primer	Sequence of primers (5′–3′)	Product size/bp
TLR2	Forward	CCT​GCT​GAC​GCT​GAA​AAA​CC	101
	Reverse	TCA​GCC​GTC​TCA​ACC​TTT​CC	
TLR4	Forward	AGA​AAT​CTG​GGA​GCC​CTG​TG	151
	Reverse	GCT​ATG​GCT​GCC​TAA​ATG​CTC	
MYD88	Forward	CCC​TTT​GTC​TCT​CGA​CTC​TTG​G	185
	Reverse	TAC​GAG​AAC​AGC​CAC​TGC​CC	
NF-κB1	Forward	ATG​CCA​ATG​CCC​TCT​TCG​ACT	164
	Reverse	CGT​GAC​TTC​CAG​CAG​ATC​CCT	
Actin	Forward	TGG​CCG​AGG​ACT​TTG​ATT​GT	170
	Reverse	TTA​CAC​AAA​TGC​GAT​GCT​GCC	

### 2.2 Establishment of the chromatogram characteristics of Zhuifeng tougu

#### 2.2.1 Test solution

##### 2.2.1.1 Test product

Approximately 2g of ZFTG was weighed accurately and placed in a conical flask with a stopper before adding 50 ml of pure water for ultrasonic treatment (power 250 W, frequency 40 kHz) for 30 min. Then, the capsules were removed, centrifuged (rotation speed: 12,000 rpm) for 10 min, and the supernatant was filtered, followed by continuous filtration.

##### 2.2.1.2 Reference product

A proper amount of paeoniflorin reference standard was accurately weighed and mixed with methanol to prepare a solution containing 20 μg per 1 ml.

#### 2.2.2 Chromatographic conditions and instrumentation

##### 2.2.2.1 Instruments

Altogether, we used a UPLC H-Class system from Waters Corporation (Milford, MA, United States), MSA3-6P-OCE-DM millionth electronic balance from Sartorius Co., Ltd. (Beijing, China), MS105DU from Mettler-Toledo Group (Shanghai, China), and KQ250-DB from Kun Shan Ultrasonic Instruments Co., Ltd. (Jiangsu, China).

##### 2.2.2.2 Chromatographic conditions

Chromatographic column: Waters HSS T3 column (2.1 mm×100 mm, 1.8 μm); mobile phase: A was acetonitrile, B was 0.1% formic aqueous acid solution; flow rate: 0.5 ml min^−1^; column temperature: 40°C; injection volume: 1 μl; detection wavelength: 230 nm; many theoretical plates: not less than 5000 according to the paeoniflorin peak; separation degree with other peaks: greater than 1.0. All components were detected within 60 min. The gradient elution process is illustrated in Table 3.

**TABLE 3:** UPLC gradient elution process.

Time (min)	Mobile phase A	Mobile phase B
0–5	2%→5%	98%→95%
5–8	5%→15%	95%→85%
8–17	15%→30%	85%→70%
17–20	30%→55%	70%→45%
20–21	55%→2%	45%→98%
21–25	2%	98%

**FIGURE 2:**
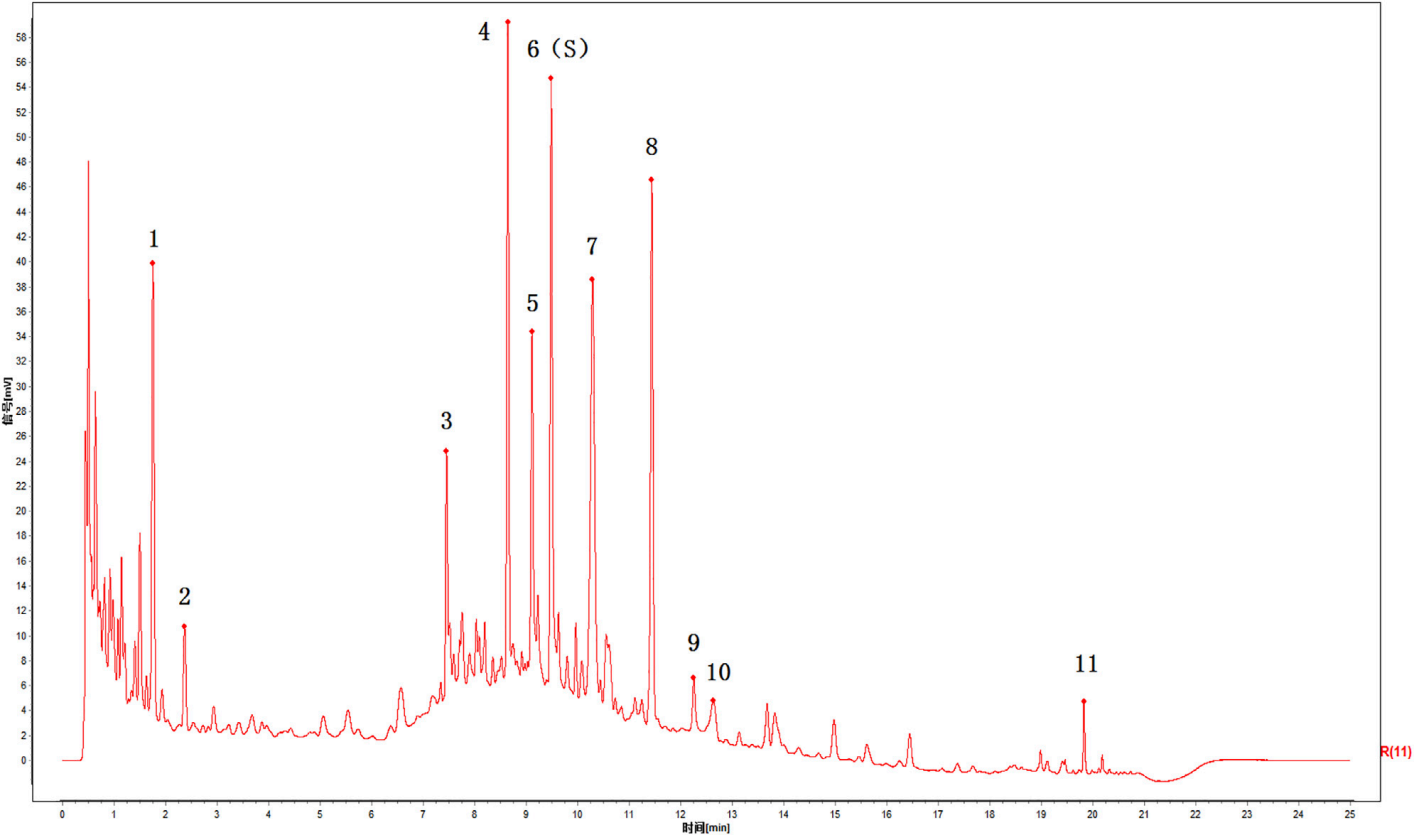
UPLC chromatograms of ZFTG. 6(S): paeoniflorin belongs to *Paeonia veitchii* Lynch. (Chi Shao, Ranunculaceae) in Northwest China, 4: *Gentiana macrophylla* Pall. (Qin Jiao, Gentianaceae) in North China, 5: *Aconitum kusnezoffii* Reichb. (Cao Wu, Ranunculaceae) in North China, 8: *Notopterygium incisum* Ting. ex H. T. Chang (Qiang Huo, Umbelliferae) in Northwest China, 9: *Saposhnikovia divaricata* (Turcz.) Schischk. (Fang Feng, Umbelliferae) in Northeast China, 10: *Ligusticum chuanxiong* Hort. (Chuan Xiong, Umbelliferae) in Sichuan China, 11: *Glycyrrhiza uralensis* Fisch. (Gan Cao, Fabaceae) in Northwest China.

According to the chromatographic conditions, six batches of ZFTG were prepared as test solutions for UPLC analysis (ZFTG lots no. 210401, 210402, 210403, 210404, 210405, and 210406) using paeoniflorin as the reference substance (lot no. 110736–202044, with 96.8% content) purchased from the National Institutes for Food and Drug Control (Beijing, China). The chromatograms were recorded for 60 min, and the UPLC fingerprints of six batches of ZFTG were obtained (Figure 2). The results were as follows: peak 6, taken as the S peak, belonging to *Paeonia veitchii* Lynch (Chi Shao, Ranunculaceae); peak 4 belonging to *Gentiana macrophylla* Pall (Qin Jiao, Gentianaceae); peak 5 belonging to *Aconitum kusnezoffii* Reichb (Cao Wu, Ranunculaceae); peak 8 belonging to *Notopterygium incisum* Ting. ex H. T. Chang (Qiang Huo, Umbelliferae); peak 9 belonging to *Saposhnikovia divaricata* (Turcz.) Schischk (Fang Feng, Umbelliferae); peak 10 belonging to *Ligusticum chuanxiong* Hort (Chuan Xiong, Umbelliferae); and peak 11 belonging to *Glycyrrhiza uralensis* Fisch (Gan Cao, Fabaceae).

### 2.3 Animals

Three-month-old New Zealand rabbits, weighing 2.0–2.5 kg, were purchased from the Animal Experiment Center of Hunan University of Chinese Medicine [animal license number: SCXK (Hunan, 2020-0005)]. All rabbits were kept in individual cages in the Animal Center Laboratory of Hunan University of Chinese Medicine [establishment license number: SYXK(Hunan, 2019-0009)]. The feeding temperature was between 24 and 26°C, and the humidity was between 50% and 70%. After 2 weeks of acclimatization with *ad libitum* access to normal water and a standard diet, the rabbits were randomly separated into two groups: the control (BC) (*n* = 9)and KOA (KOA) (*n* = 33). To generate a model of OA, we chose the modified Videman method (Liu J et al., 2021) and the left hindlimb, which was immobilized for 6 weeks with a plaster cast to replicate the KOA model.

Six weeks after modeling, three rabbits were randomly selected from the BC or KOA groups for KOA model validation, according to the random number table method. After model validation, the KOA model rabbits were randomly divided into five groups: model control (MC, KOA rabbits + saline); high-dose (HD, KOA rabbits + high-dose ZFTG, 112 mg/kg body weight); medium-dose (MD, KOA rabbits + medium-dose ZFTG, 56 mg/kg body weight); low-dose (LD, KOA rabbits + low-dose ZFTG, 28 mg/kg body weight); or positive control groups (PC, KOA rabbits + GS 50 mg/kg body weight), with a total of 30 rabbits (six rabbits in each group). The control group (BC, *n* = 6) received saline gavage (10 ml/kg body weight). The gavage dose of ZFTG and GS in rabbits was equivalent to that used in patients.

After 6 weeks of gavage, the molded side knee joint was removed for observation and pathological staining. A subsample was stored in a cryopreservation tube at −80 °C for quantitative real-time polymerase chain reaction (qRT-PCR) and western blotting (WB) analyses. Individuals were euthanized according to the IAEC animal experimentation guidelines. All experimental protocols were approved by the Committee of Ethics on Animal Experiments at the Hunan University of Chinese Medicine (LLBH-202007070001).

### 2.4 Model validation

The validation method included comparing the functional activities and general observation of knee cartilage to assess cartilage damage. After the general observation, frozen sections of the knees (5 mm thick) were collected and observed under a microscope using a modified 100 and 400 OA Research Society International (OARSI) scoring system (Waldstein et al., 2016). The OARSI system, widely used to evaluate articular cartilage lesions in animals and humans, can identify early or moderate OA lesions.

### 2.5 Histology and immunohistochemistry

All collected sections were dewaxed with dimethylbenzene, soaked in graded ethyl alcohol, and washed with distilled water. Parts of the sections were stained with hematoxylin and eosin (H&E), dehydrated with gradient alcohol (95%–100%), and sealed. Urea and trypsin antigen were used to repair other parts of the sections, which were subsequently treated with primary and secondary antibodies, and then DBA was added for 5 min to develop color and observed with a microscope. Other sections were immersed in EDTA buffer (pH 9.0), heated, and then cooled to room temperature. Before adding the primary and secondary antibodies, the sample was first cleaned with 0.01M PBS (pH 7.4–7.6), submerged in 75% alcohol, Sudan black dye solution was added, and then stained with DAPI working solution at 37°C until sealed. The H&E staining solution and immunohistochemistry reagents and kits were purchased from Wellbio (K435960 and K484350) and Beijing ZSGB company (600D54 and 600W23), respectively. The immunofluorescence reagents were purchased from ProteinTech (SA00013-2). The stained cartilages were observed and imaged using a light microscope (BA410T, Motic China Group Co., Ltd., Xiamen, China). Immunohistochemical sections were examined for IL-1β, IL-6, TNF-α, and IFN-γ. Immunofluorescence sections were used to observe the expression of NF-κB p65.

### 2.6 Cell cultures

Primary chondrocytes were obtained from rabbit knee cartilage. Chondrocytes were maintained in 10% Dulbecco’s modified Eagle’s medium (DMEM). After digesting the cells with trypsin, adherent cells were collected and subcultured. Chondrocytes from passage 3 (P3) were used for further analysis. The P3 generation of chondrocytes was randomly divided into nine groups and then treated with different concentrations of ZFTG (0, 50, 100, 200, 400, 800, 1000, 1600, and 2000 ng/μl) to select the best concentration.

The chondrocytes were modeled with 10 ng/ml IL-1β and grown on glass slides fixed with 4% paraformaldehyde. Inactivated endogenous enzymes were identified with toluidine blue and collagen type II (Rabbit ab34712, Abcam, Cambridge, United Kingdom) for immunocytochemical staining under microscopic observation. The P3 generation of chondrocytes was randomly divided into five groups. In addition to the control group, the other groups were treated with IL-1β and then treated with different concentrations of ZFTG (0, 100, 200, and 400 ng/μl). The selection of these concentrations was based on the screening results of the previous drug concentration.

### 2.7 Cytotoxicity and apoptosis

The Cell Counting Kit-8 (CCK-8) was used to assess the cytotoxicity of various concentrations of ZFTG against the P3 generation of chondrocyte rabbits. Cells were seeded in 96-well plates at a density of 10^4^ cells/well, followed by treatment with IL-1β and various ZFTG concentrations at the indicated dosages for 24 h. After cell intervention for 24 h, 20 μl CCK-8 solution was added into each well and incubated for 4 h in a 5% CO_2_ incubator at 37°C. The optical density (OD) values at 450 nm were measured using a microplate reader (DSZ 2000X, Zhongxian Hengye Corp, Beijing, China).

The apoptosis of P3 chondrocytes treated with IL-1β (10 ng/ml) and various ZFTG was measured using flow cytometry. Cells were digested with 0.25% trypsin, which contained 0.02% EDTA, before collecting the cell suspension. The cells were collected after the suspension was centrifuged for 5 min (1500 rpm), washed with PBS, and mixed with Annexin V-FITC and propidium iodide. Annexin V was subjected to fluorescein FITC labeling of apoptotic chondrocytes using the Annexin V-FITC apoptosis detection kit (KGA108, KeyGen Biotech, Nanjing, China).

### 2.8 Western blot analysis

*In vivo* and *in vitro* experiments of the cartilage tissue weighing approximately 0.025 g were carried out, and chondrocytes were precooled, washed with ice and PBS, and crushed with 300 µl RIPA lysate in a biological sample homogenizer, and the complete protein was extracted. The protein supernatant was mixed with loading buffer, boiled in water for 5 min, and placed in an ice box for medium-speed cooling. BCA detection (AWB0104, Abiowell, China) was used to carry out protein quantification, and electrophoresis was carried out for 130 min according to the results. After blocking with 5% non-fat-dried milk at room temperature for 1 h, membranes were incubated with primary antibodies overnight at 4°C, and then incubated with horseradish peroxide-conjugated secondary antibodies at room temperature for 90 min. The ECL reagent (K-12045-D50, Advansta, CA, United States) was incubated with the membrane for 1 min, and the exposure was performed in the chemiluminescence imaging system (ChemiScope6100, Clinx, Guangzhou, China).

### 2.9 Real-time polymerase chain reaction

The total RNA was extracted with TRIzol reagent (15596026, Thermo Fisher Scientific, CA, United States) according to the manufacturer’s instructions. Total mRNA was used as a template for reverse transcription of cDNA (CW2141, Beijing ComWin Biotech Co., Ltd., China) following the manufacturer’s protocol. Reverse transcription products were retained for PCR reactions and fluorescence quantitative PCR reactions. Each sample was analyzed in triplicate, and expressions of TLR2, TLR4, MyD88, and NF-κB were normalized to the expression level of β-actin. The 2^−ΔΔCt^ method (Li et al., 2014) was used to calculate the relative expression levels of each gene.

### 2.10 Statistical analysis

All data were expressed as the mean ± standard deviation. Data from two groups were compared using the Mann–Whitney test. A comparison among three or more groups was performed using one-way analysis of variance (ANOVA). SPSS for Windows (version 26) was used to analyze the data. The graphs were plotted using GraphPad Prism (version 8). A *p* < 0.05 was considered statistically significant. To reduce the uncertainty and contingency of the data, we reported the mean difference between groups and the upper and lower limits of the 95% confidence interval.

## 3 Results

### 3.1 *In vivo* experiments

#### 3.1.1 Knee osteoarthritis model establishment

**FIGURE 3:**
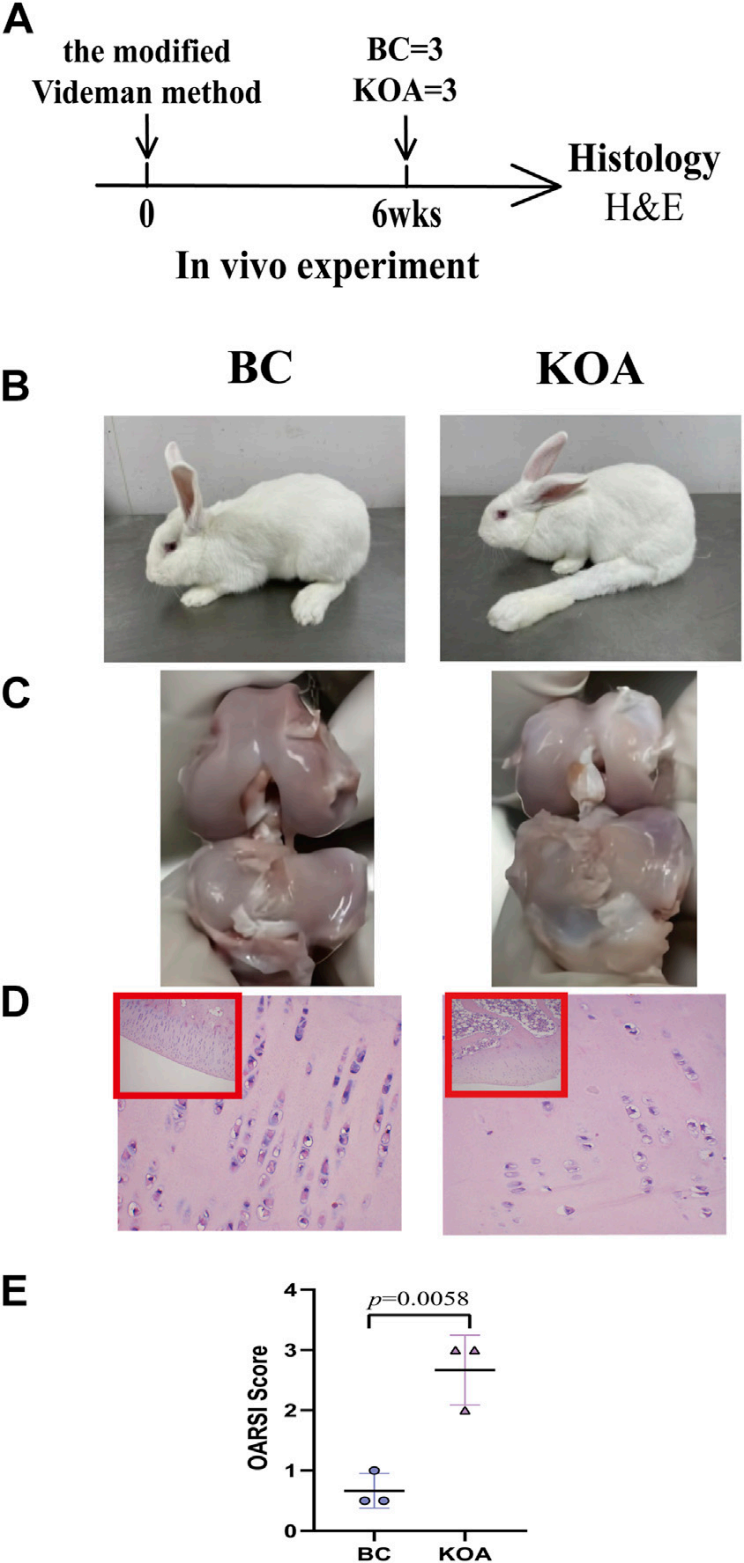
KOA model validation. **(A)** Experimental design. The KOA model rabbits were treated with the modified Videman modeling. The rabbits were killed 6 weeks after surgery for macroscopic observation and histological analysis (*n* = 3/group). **(B)** Outward and **(C)** anatomical appearance of rabbit knee joint. **(D)** Representative images and **(E)** histomorphometric analyses of H&E-stained sections for OARSI score. The images are magnified 100×(inside the red rectangular frame) and 400×. Mann–Whitney test was used for the comparing OARSI scores. The data are presented as the mean ± SD. Specific *p-*values of comparison are reported.

According to the experimental design, some rabbits were treated with modified Videman modeling at 3 months. To verify the success of the modified Videman modeling, we randomly selected three experimental rabbits from the BC and KOA groups for model verification 6 weeks later (Figure 3A). In the BC group, the outward and flexion–extension functions of the knee joints were normal. The left knee joint of the model group was stiff, and the joint flexion activity was limited (Figure 3B). The anatomical appearance of the left knee joint in the BC group rabbits revealed complete, smooth, and lustrous cartilage, whereas the KOA model group demonstrated that the cartilage exhibited a surface without luster, a thinner layer and a rough surface, and the cartilage in the bearing area of the joint surface was broken or with downward ulceration (Figure 3C). H&E staining of the articular cartilage in the BC group demonstrated that the cells in the surface and deep layers were arranged neatly and presented a normal shape, without pathological manifestation. In the model group, the cartilage layer became thinner, the chondrocytes were absent and distributed irregularly, and tidal lines were disordered, which was consistent with the early pathological changes of OA (Figure 3D). H&E-stained sections (Figure 3E) revealed that KOA model rabbits had more severe joint damage than BC rabbits, evidenced by higher OARSI scores (group difference: 2.0003, 95% CI: 0.9653 to 3.035, *p* = 0.0058). All the abovementioned results indicate that the modeling in the KOA group was successful.

#### 3.1.2 Zhuifeng tougu improves OA

**FIGURE 4:**
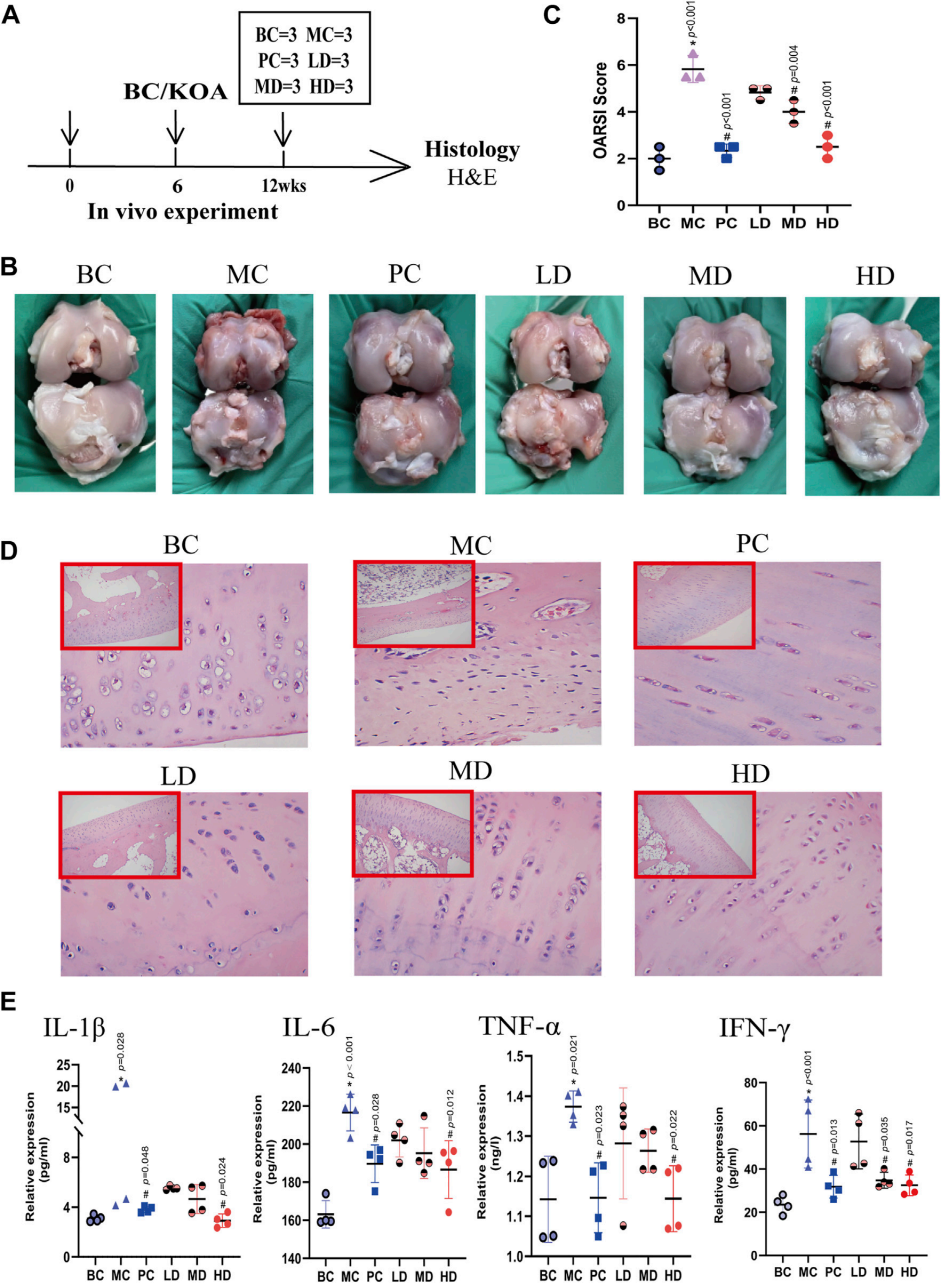
Effect of ZFTG on KOA rabbit. **(A)** Experimental design. The rabbits were killed for H&E analysis at 12 weeks (*n* = 3/group). **(B)** Anatomical appearance of rabbit knee joint. **(D)** Representative images and **(C)** histomorphometric analyses of H&E-stained sections for OARSI score. The images are magnified 100× (inside the red rectangular frame) and 400×. **(E)** IL-1β, IL-6, TNF-α, and IFN-γ levels were measured by the ELISA kit according to the manufacturer’s instructions. One-way analysis of variance was used for comparing the OARSI scores. The data are presented as mean ± SD. *p < 0.05, vs. BC group; #*p* < 0.05, vs. MC group. Specific *p*-values for each pair of comparisons are reported.

**FIGURE 5:**
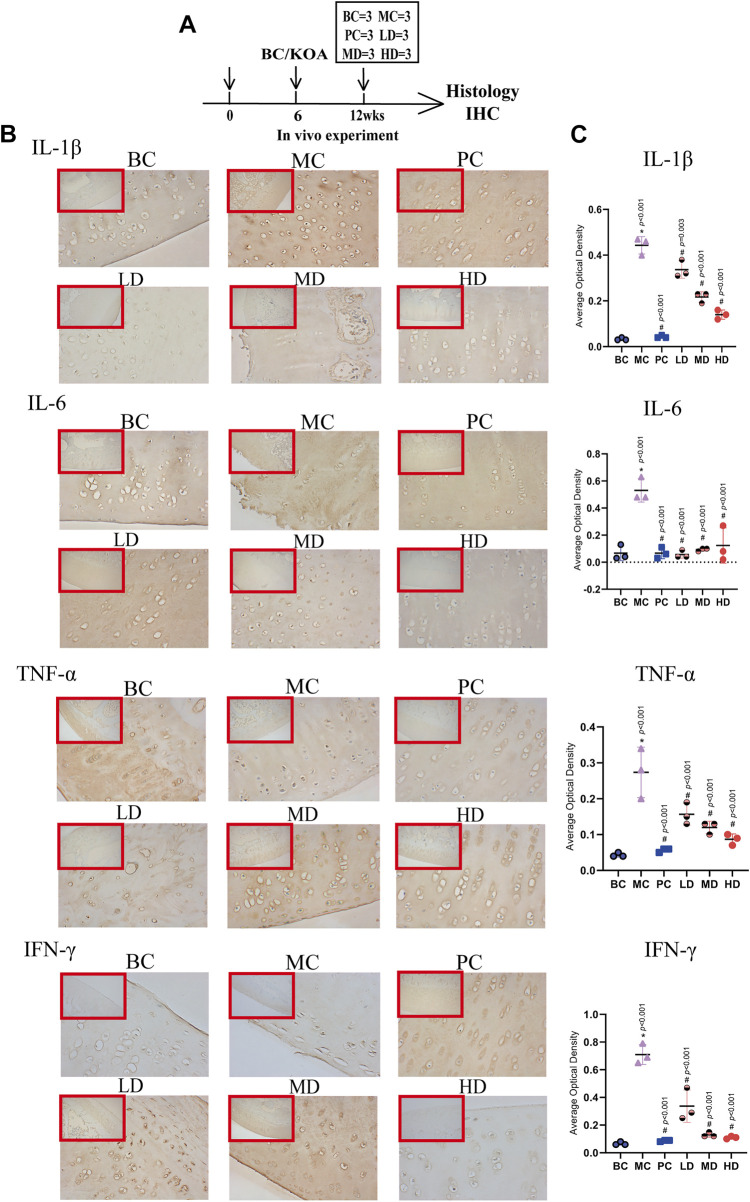
Immunohistochemistry of IL-1β, IL-6, TNF-α, and IFN-γ. **(A)** Experimental design. The rabbits were killed for immunohistochemical analysis at 12 weeks (*n* = 3/group). (B) Representative images and **(C)** average optical density (AOD) analyses of immunohistochemical sections IL-1β, IL-6, TNF-α, and IFN-γ. The images are magnified 100× (inside the red rectangular frame) and 400×. One-way analysis of variance was used for comparing AOD scores. The data are presented as the mean ± SD. *p < 0.05, vs. BC group; #*p* < 0.05, vs. MC group. Specific *p-*values for each pair of comparisons are reported.

After successful modeling, the rabbits in each group were treated intragastrically according to the experimental plan. Six weeks after dosing, all rabbits were killed, and the left knee joint was collected for anatomical (Figure 4A) and histological examination (Figure 5A).

Macroscopically, we found that the knee joints of the MC group were more worn than those of the BC group and manifested as grayer, thinner, and lusterless cartilage. Because the cartilage in the bearing area of the articular surface was damaged, the articular surface was unsmooth and even dented downward, forming ulcers. Moreover, osteophytes were formed. The cartilage of the four medication groups (i.e., PC, LD, MD, and HD) was better than that of the MC. In the ZFTG groups, especially the HD, the cartilage tissue was less worn, and the functional performance was better. The articular cartilage in the HD group was yellowish, more lustrous, and thicker than that in the MC, and a slight abrasion was observed in the mid-posterior region of the medial tibial plateau without significant pitting or osteophyte formation (Figure 4B). The H&E-stained sections revealed that the MC group had more severe joint damage than the BC group, and thinner cartilage layers, with fewer but more disorganized chondrocytes. However, the cartilage condition in the ZFTG groups was better than that in the MC (Figure 4D), as evidenced by the OARSI scores (Figure 4C).

#### 3.1.3 Zhuifeng tougu relieves inflammatory reaction of OA

To explore how ZFTG relieves joint injury, we conducted a series of tests on the blood and cartilage samples of experimental rabbits according to the previous investigation (Gong et al., 2021). To verify the anti-inflammatory effect of ZFTG, we measured the levels of IL-1β, IL-6, TNF-α, and IFN-γ in the serum and articular cartilage of rabbits in each group. Serum test results (Figure 4E) demonstrated that the expression of each factor in the MC group [group difference: IL-1β (9.264, 95% CI: 0.7616 to 17.77, *p* = 0.028), IL-6 (53.51, 95% CI: 28.79 to 78.22, *p* < 0.001), TNF-α (0.2314, 95% CI: 0.0274 to 0.4355, *p* = 0.021), and IFN-γ (32.72, 95% CI: 12.41 to 53.03, *p* < 0.001)] increased significantly than that in the BC group. The MC group presented inflammatory manifestations.

The expression levels of related factors in the ZFTG groups were lower than those in the MC, and the resultant trends were consistent with those in the PC group. Among these, IL-1β (−9.437, 95% CI: −17.94 to −0.9345, *p* = 0.024), IL-6 (−30.00, 95% CI: −54.72 to 5.278, *p* = 0.038), TNF-α (−0.2298, 95% CI: −0.4339 to −0.0258, *p* = 0.022), and IFN-γ (−23.73, 95% CI: −44.04 to −3.419, *p* = 0.017) of the HD group had lower expression levels than those in the MC group, but the LD and MD groups had no statistical significance compared to the MC group. The abovementioned results indicated that a high-dose ZFTG could significantly inhibit inflammation by reducing IL-1β, IL-6, TNF-α, and IFN-γ in the serum of rabbits with OA. Immunohistochemical results of articular cartilage sections are demonstrated in (Figure 5B). Moreover, we used ImageJ software to quantitatively study the average optical density (AOD) value in cartilage slices to represent the expression of each factor (Figure 5C). The expression of each factor in the model groups [group difference: IL-1β (0.4100, 95% CI: 0.3404 to 0.4796, *p* < 0.001), IL-6 (0.4633, 95% CI: 0.2689 to 0.6578, *p* < 0.001), TNF-α (0.2300, 95% CI: 0.1400 to 0.3200, *p* < 0.001), and IFN-γ (0.6433, 95% CI: 0.4870 to 0.7997, *p* < 0.001)] were significantly higher than that in the normal group. The results demonstrated that the model group had obvious inflammatory manifestations in the articular cartilage, consistent with the serum test results.

However, the expression levels of inflammatory factors in ZFTG groups decreased, which had the same trend as the results of the positive drug group. IL-1β (−0.3033, 95% CI: −0.3730 to −0.2337, *p* < 0.001), IL-6 (−0.4067, 95% CI: −0.6011 to −0.2122, *p* < 0.001), TNF-α (−0.1867, 95% CI: −0.2767 to −0.0966, *p* < 0.001), and IFN-γ (−0.6000, 95% CI: −0.7563 to −0.4437, *p* < 0.001) of the HD group were significantly lower than those in the MC group. Based on the results of the abovementioned blood and cartilage tests, we found that ZFTG can reduce the expression of inflammatory factors in the body and cartilage, thereby relieving the inflammatory response of KOA. This was also confirmed by the anatomical observation of the knee joint and the results of the H&E-stained section.

#### 3.1.4 Zhuifeng tougu alleviates knee osteoarthritis in rabbits by inhibiting the TLR4/MyD88/NF-κB signaling pathway

**FIGURE 6:**
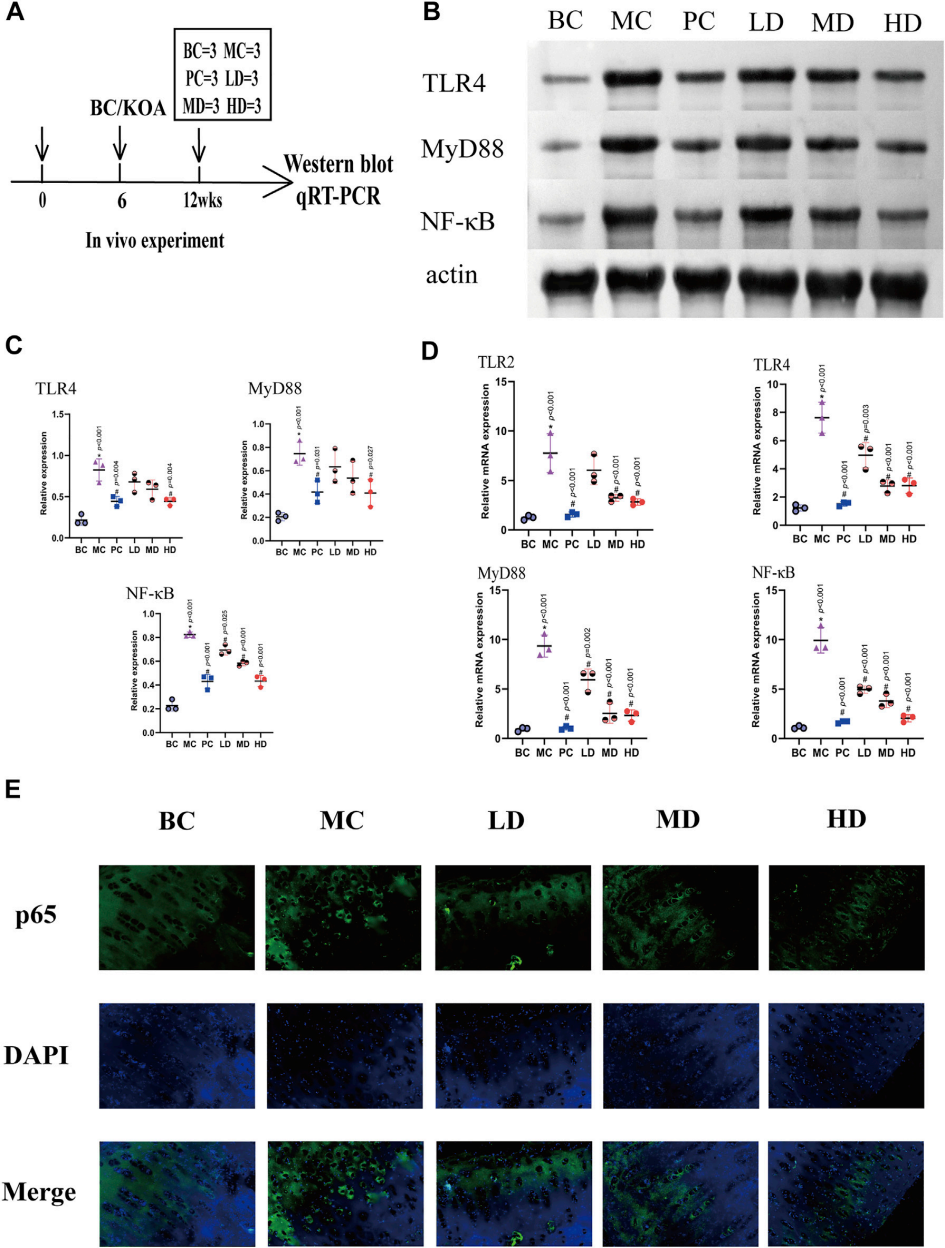
ZFTG inhibits the TLR2-4/MyD88/NF-kB signaling pathway in alleviating knee osteoarthritis in rabbits. **(A)** Experimental design. The rabbits were killed for Western blot and qRT-PCR analysis at 12 weeks (*n* = 3/group). **(B,C)** Western blot analysis of TLR4, MyD88, and NF-kB protein expression with actin serving as a protein loading control. **(D)** qRT-PCR analysis of TLR2, TLR4, MyD88, and NF-kB mRNA expression. **(E)** Immunofluorescence observation of NF-κB p65 protein expression. The images are magnified 400×. One-way analysis of variance was used for comparing protein and gene expression. The data are presented as the mean ± SD. *p < 0.05, vs. BC group; #*p* < 0.05, vs the MC group. Specific *p*-values for each pair of comparisons are reported.

To further explore the anti-inflammatory effect of ZFTG, we studied the mechanism by detecting the protein and mRNA levels of TLR4, MyD88, and NF-κB in the articular cartilage (Figure 6A).

The expression of the TLR4/MyD88/NF-κB signaling pathway would induce differential expression of the knee joint. The protein expression is demonstrated in Figures 6B,C, and the expression levels of the protein in the MC group were significantly higher than those in the BC [group difference: TLR4 (0.6067, 95% CI: 0.3450 to 0.8684, *p* < 0.001), MyD88 (0.5400, 95% CI: 0.2353 to 0.8447, *p* < 0.001), and NF-κB (0.5967, 95% CI: 0.4805 to 0.7128, *p* < 0.001)]. The test results in the ZFTG groups were consistent with those in the PC group and were lower than those in the MC group. With the increase in ZFTG concentration, the corresponding expressions of TLR4, MyD88, and NF-κB proteins decreased gradually. The protein expressions of TLR4 (−0.3800, 95% CI: −0.6417 to −0.1183, *p* = 0.004), MyD88 (−0.3367, 95% CI: −0.6414 to −0.0320, *p* = 0.027), and NF-κB (−0.3900, 95% CI: −0.5062 to −0.2738, *p* < 0.001) in the HD group were significantly different from those in the MC group. The protein expression of NF-κB in LD and MD groups was significantly different from that in the MC group [(LD, −0.1300, 95% CI: −0.2462 to −0.0138, *p* = 0.025), (MD, −0.2400, 95% CI: −0.3562 to −0.1238, *p* < 0.001)]. However, there was an insignificant difference in the protein expression of TLR4 and MyD88.

The abovementioned results indicated that the expression levels of TLR4, MyD88, and NF-κB proteins varied with the development of the disease. To verify this trend and confirm the role of the TLR signaling pathway in relieving KOA with ZFTG, we further observed the mRNA expression of TLR2, TLR4, MyD88, and NF-κB in articular cartilage. The results are displayed in Figure 6D. The mRNA expressions in the MC group were higher than those in the BC group [group difference:TLR2 (6.521, 95% CI: 3.713 to 9.329, *p* < 0.001), TLR4 (6.401, 95% CI: 4.613 to 8.190, *p* < 0.001), MyD88 (8.420, 95% CI: 6.225 to 10.61, *p* < 0.001), and NF-κB (8.823, 95% CI: 7.076 to 10.570, *p* < 0.001)]. It was consistent with the protein expression results of cartilage proteins. Compared with the MC group, the mRNA expression level of the PC group decreased significantly. Resembling the PC group, the mRNA expression in ZFTG groups was significantly lower than that of the MC group. Similar to the positive control group, the level of serum TNF-α in ZFTG groups was significantly lower than that in the model, and the HD group demonstrated the lowest expression of mRNA [group difference:TLR2 (−4.935, 95% CI: −7.742 to −2.127, *p* < 0.001), TLR4 (−4.817, 95% CI: −6.606 to −3.029, *p* < 0.001), MyD88 (−7.028, 95% CI: −9.223 to −4.833, *p* < 0.001), and NF-κB (−7.891, 95% CI: −9.638 to −6.144, *p* < 0.001)]. The NF-κB p65 protein was evenly expressed in the cytoplasm of the BC group, but the MC group showed significantly higher levels in the nucleus. The expression of NF-κB p65 protein in the nucleus gradually reduced following intervention with ZFTG (Figure 6E). These data indicate that the inflammatory response caused by OA is closely related to the TLR signaling pathway. ZFTG not only inhibited the TLR2-4/MyD88/NF-κB signaling pathway but also effectively relieved the development of OA.

### 3.2 *In vitro* experiment

Based on the significant efficacy of the drugs in the animal experiments, we conducted additional *in vitro* studies.

#### 3.2.1 Zhuifeng tougu enhances the activity of rabbit chondrocytes

**FIGURE 7:**
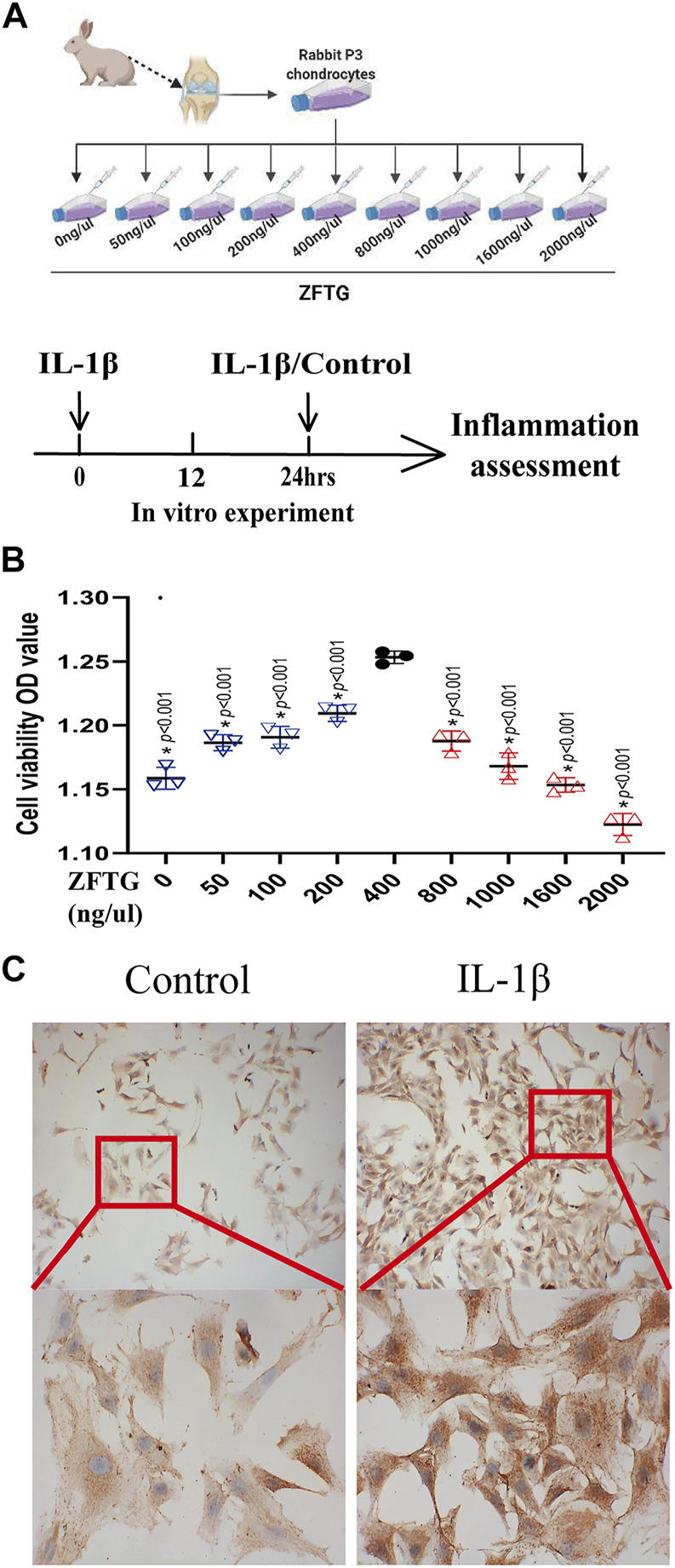
ZFTG enhances the activity of rabbit chondrocytes. **(A)** Experiment design for the cell. Primary chondrocytes obtained from rabbit knee cartilage (New Zealand rabbits, male, 3-month-old), were treated with ZFTG (0, 50, 100, 200, 400, 800, 1000, 1600, and 2000 ng/μl) to evaluate cell viability. Cells were treated with IL-1β (10 ng/ml) for 24 h s (M_IL-1β_) to assess inflammation. **(B)** Cell viability after ZFTG intervention at various concentrations. (C) Expression of collagen II in chondrocytes of the control and IL-1β groups. The images are magnified 100× and 400×. One-way analysis of variance was used for comparing the cell viabilities. The data are presented as the mean ± SD. *p < 0.05, vs. the 400 ng/uL ZFTG group. Specific *p*-values for each pair of comparisons are reported.

To verify the effect of ZFTG on rabbit chondrocytes, we used different concentrations of ZFTG solution (Figure 7A). The results demonstrated (Figure 7B) that chondrocytes were the most active at a concentration of 400 ng/μl. As the concentration of ZFTG was higher than 400 ng/μl, the activity of chondrocytes was gradually decreased; thus, we selected the 400 ng/μl ZFTG as the standard concentration for subsequent experiments.

#### 3.2.2 Establishment of an inflammatory injury model of chondrocytes

We treated chondrocytes with IL-1β for 24 h to induce a pro-inflammatory phenotype (McNulty et al., 2013), and the expression of collagen II was used as the criterion to verify the model (Figure 7A). As displayed in Figure 7C, the expression of collagen II in chondrocytes in the IL-1β group was significantly higher than that in the control group, which demonstrated that inflammatory injury in chondrocytes was successfully established.

#### 3.2.3 Effect of Zhuifeng tougu on chondrocyte injury induced by inflammation

**FIGURE 8:**
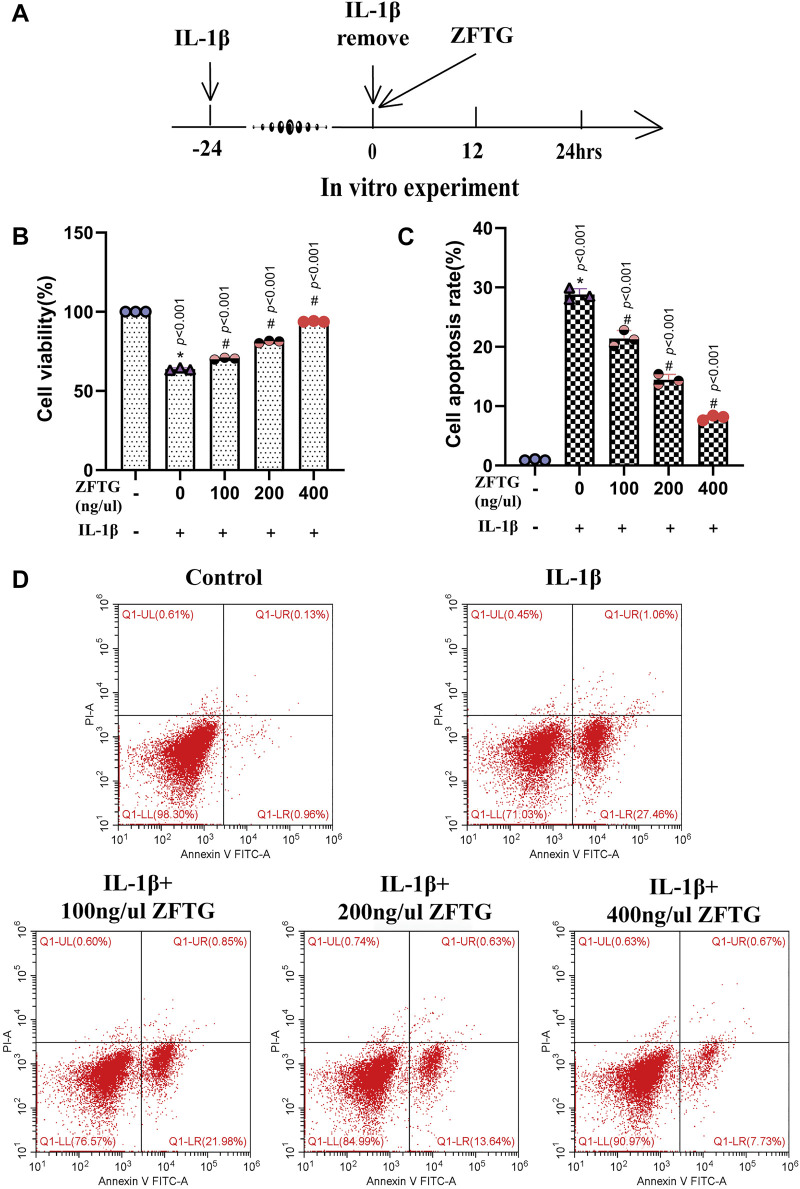
ZFTG improves inflammatory injury of cartilage cells. **(A)** Experiment design for cells. Primary chondrocytes were stimulated with IL-1β (10 ng/ml) for 24 h s (M_IL-1β_) before removing IL-1β and adding ZFTG (0, 100, 200, and 400 ng/μl) treatment to evaluate cell viability and apoptosis rate. Cells were harvested 24 h after ZFTG treatment. **(B)** Cell viability after ZFTG intervention at various concentrations based on the control group (100%). **(C)** Cell apoptosis rate after ZFTG intervention at various concentrations. **(D)** Apoptosis of chondrocytes among five groups by flow cytometry. One-way analysis of variance was used for comparing the cell viabilities and apoptosis rates. The data are presented as the mean ± SD. *p < 0.05, vs. the control group; #*p* < 0.05, vs. IL-1β group. Specific *p*-values for each pair of comparison are reported. The control group: ZFTG^−^/IL-1β^−^; the IL-1β group: ZFTG^−^/IL-1β^+^.

We treated the chondrocytes with ZFTG and detected cell survival (Figure 8A). The results demonstrated that (Figures 8B–D) the control group had significantly higher cell viability than the IL-1β group (group difference: 36.23, 95% CI: 34.73 to 37.72, *p* < 0.001) and lower apoptotic rate (group difference: −27.86, 95% CI: −30.20 to −25.51, *p* < 0.001), suggesting that the intra-articular inflammation in OA patients was indeed closely related to the wear of articular cartilage. However, ZFTG could significantly affect the activity of the damaged chondrocytes and inhibit their apoptosis. The best ZFTG concentration was 400 ng/μL (compared with the IL-1β group: cell viability: 30.06, 95% CI: 28.56 to 31.56, *p* < 0.001; cell apoptosis rate: −20.74, 95% CI: −23.09 to −18.39, *p* < 0.001), which was similar to the previous experimental result.

**FIGURE 9:**
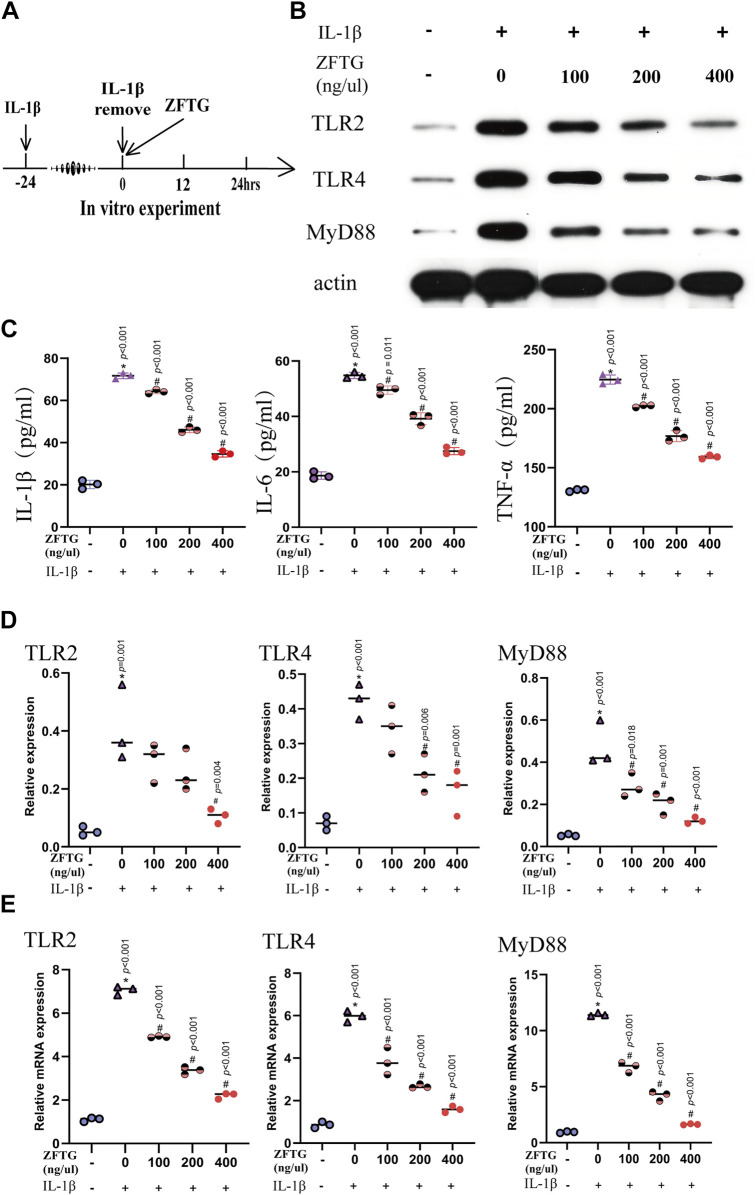
ZFTG inhibits the TLR2/TLR4/MyD88 signaling pathway in alleviating inflammatory damage of chondrocytes. **(A)** Experiment design for cells. Primary chondrocytes were stimulated with IL-1β (10 ng/ml) for 24 h s (M_IL-1β_) before removing IL-1β and adding ZFTG (0, 100, 200, and 400 ng/μl) treatment. **(B,D)** Western blot analysis of TLR2, TLR4, and MyD88 protein expression with actin serving as a protein loading control. **(C)** IL-1β, IL-6, and TNF-α levels were measured by the ELISA kit according to the manufacturer’s instructions. **(E)** qRT-PCR analysis of TLR2, TLR4, and MyD88 mRNA expression. One-way analysis of variance was used for comparing the protein and gene expression. The data are presented as the mean ± SD. *p < 0.05, vs. BC group; #*p* < 0.05, vs. MC group. Specific *p*-values for each pair of comparisons are reported.

Moreover, we detected the expression of inflammatory factors in cell supernatants. As displayed in Figure 9C, the expressions of factors in the IL-1β group were significantly higher than those in the control group [IL-1β (51.53, 95% CI: 47.59 to 55.46, *p* < 0.001), IL-6 (36.16, 95% CI: 32.05 to 40.27, *p* < 0.001), and TNF-α (93.83, 95% CI: 86.20 to 101.5, *p* < 0.001].The intervention of ZFTG reduced the expression of factors to varying degrees, among which, the 400-ng/μl ZFTG groups had the lowest expression [IL-1β (−37.05, 95% CI: −40.99 to −33.11, *p* < 0.001), IL-6 (−27.33, 95% CI: −31.44 to −23.21, *p* < 0.001), and TNF-α (−65.58, 95% CI: −73.21 to −57.96, *p* < 0.001)]. Therefore, ZFTG had a stable anti-inflammatory effect on chondrocytes, which was consistent with the results of the previous experiment.

#### 3.2.4 Zhuifeng tougu inhibits the TLR2/TLR4/MyD88 signaling pathway and mitigates inflammatory injury of cartilage cells

To further verify the anti-inflammatory mechanism of ZFTG on chondrocytes, we detected the proteins and mRNA related to TLR2, TLR4, and MyD88 in chondrocytes as in the *in vivo* experiment (Figure 9A).

As demonstrated in Figures 9B,D, the expression levels of TLR2, TLR4, and MyD88 proteins were highest in the IL-1β group [compared to the control group: TLR2 (0.3567, 95% CI: 0.1540 to 0.5593, *p* < 0.001), TLR4 (0.3533, 95% CI: 0.2045 to 0.5021, *p* < 0.001), and MyD88 (0.4233, 95% CI: 0.2641 to 0.5826, *p* < 0.001)]. These data suggest a positive correlation between the severity of OA and expression of TLR2, TLR4, and MyD88 proteins. With the increase of drug concentration, the protein expressions of TLR2, TLR4, and MyD88 in ZFTG groups decreased gradually, and the best ZFTG concentration was 400 ng/μl [compared to the IL-1β group:TLR2 (−0.3033, 95% CI: −0.5060 to −0.1007, *p* < 0.001), TLR4 (−0.2600, 95% CI: −0.4088 to −0.1112, *p <* 0.001), and MyD88 (−0.3533, 95% CI: −0.5126 to −0.1941, *p* < 0.001)]. TLR2, TLR4, and MyD88 mRNA expressions in each group were consistent with the protein expression trend. The results revealed (Figure 9E) that mRNA expression levels of TLR2, TLR4, and MyD88 in the IL-1β group were the highest [compared to the control group:TLR2 (5.950, 95% CI: 5.578 to 6.323, *p* < 0.001), TLR4 (5.106, 95% CI: 4.237 to 5.976, *p* < 0.001), MyD88 (10.49, 95% CI: 9.709 to 11.28, *p* < 0.001)]. ZFTG could significantly reduce gene expression. TLR2, TLR4, and MyD88 mRNA expression levels were lowest in the 400 ng/μl group [compared to IL-1β group:TLR2 (−4.852, 95% CI: −5.224 to −4.479, *p* < 0.001), TLR4 (−4.379, 95% CI: −5.249 to −3.510, *p* < 0.001), MyD88 (−9.795, 95% CI: −10.58 to −9.010, *p* < 0.001)]. Furthermore, with the decrease in drug concentration, the inhibition of ZFTG on TLR2, TLR4, and MyD88 genes weakened gradually and manifested as a gradual increase in gene level in each group.

These data indicate that ZFTG can reduce chondrocyte inflammation, inhibit chondrocyte apoptosis, and repress the expression of genes and proteins of TLR2, TLR4, and MyD88.

## 4 Discussion

As an age-related joint degeneration, OA has been treated with general therapy (lifestyle changes (e.g., weight control and diet control), exercise plans (e.g., muscle exercise), and medication (e.g., non-steroidal anti-inflammatory drugs, analgesic drugs) with existing clinical treatment strategies that aggravate the burden and risk on the gastrointestinal tract and kidneys (Grover and Samson, 2016; Jayakumar et al., 2019; Bisson et al., 2020). In addition, there are alternative therapeutic therapies such as anti-cytokines and inhibitors [e.g., inducible nitric oxide synthase (iNOS)] or NF-κB signaling (Marcu et al., 2010) and intra-articular injection of hyaluronic acid, glucosamine, or glucocorticoid that although provide acceptable short-term effects, yet the long-term effects are not ideal (Jevsevar et al., 2015).

The occurrence and development of OA involve complex networks of inflammatory mediators. The imbalanced remodeling driven by inflammatory mediators is an important factor in inducing and promoting the development of OA (Loeser et al., 2012). Inflammation stimulates the activation of “sleeping chondrocytes,” which are “quiescent” in the low turnover state of the cartilage matrix and affect the balance between anabolism and catabolism by expressing cytokines and other genes that regulate inflammatory and catabolic responses. The inflammatory and stromal cells (chondrocytes and synovial cells) can communicate, affecting the turnover of the extracellular matrix in the joint tissue more frequently and aggravating OA (Ahmad et al., 2019).

The TLRs and NF-κB signaling pathways are important for inflammatory expression and activation of chondrocytes and are highly expressed in chondrocytes stimulated by inflammation (Geurts et al., 2011). In particular, TLR2 and TLR4 are highly expressed in the lesion, which is consistent with our study. The high expression of TLRs induces the release of pro-inflammatory and catabolic factors and triggers the loss of cartilage proteoglycans, thus aggravating OA (Bisson et al., 2020). Therefore, inhibiting the progression of inflammatory lesions in OA has become a direction to explore an effective treatment of OA (Orlowsky and Kraus, 2015).

Recently, the treatment of OA with TCM has been frequently reported, and its curative effect has been gradually recognized (Yang et al., 2017). Its effect mainly relieves OA symptoms and delays cartilage wear (Yao et al., 2021); it also has the advantages of a stable curative effect, low cost, and small side effects. In addition to internal and external administration of Chinese medicine, effective treatments of OA have been reported for needle therapy (Mao and Kapur, 2010; Fu et al., 2020), moxibustion (Wang et al., 2020), Tai chi (Chen et al., 2021; Zheng et al., 2021), baduanjin (Li J et al., 2020), and other non-drug treatments (Song et al., 2021). However, insignificant effects over the corresponding placebo have been reported in reducing pain or improving function in patients with KOA (Lao et al., 2015). Therefore, treating OA with TCM presents potential cure that cannot be ignored. Nevertheless, TCM prescriptions have complex and diversified components, so the efficacy mechanism still needs to be deeply explored (Li et al., 2009; Ziadlou et al., 2019). The composition of ZFTG varies (Gong et al., 2021). However, its mature automatic production and detection and extraction technology for the toxic substance, aristolochic acid I (A), ensures the stability and safety of the drug (see patents CN 215133530 U, CN1116503316 A, and CN 107991419 A for details). ZFTG can effectively regulate the levels of inflammatory indicators such as CRP in the blood of rats by mediating the TLR2/4-NF-κB signaling pathway and relieving the symptoms of arthritis in the feet of rats with rheumatoid arthritis (Gong et al., 2021).

The modified Videman method was a classic OA model for New Zealand rabbits (Videman, 1982). The pathological results demonstrated that the wear of articular cartilage and growth disorder of chondrocytes in the KOA group confirmed the successful modeling in this study. Treatment with IL-1β to induce chondrocytes to a pro-inflammatory phenotype is also the current general method for OA experiments *in vitro* (Blasioli and Kaplan, 2014), which also confirms edgewise the pivotal function of IL-1β in the occurrence and development of OA.

In addition, IL-1β, IL-6, and TNF-α are closely related to degeneration of articular cartilage matrix (Kapoor et al., 2011). IL-1β and TNF-α are the main participants, and they can work together and supplement each other. IL-1β, belonging to the IL-1 family, mediates the innate immune response and is related to cartilage destruction, triggering the apoptosis of chondrocytes (López-Armada et al., 2006) activated by a specific IL-11 receptor (IL-1R1) (Boraschi et al., 1996). The increased expression of the IL-1R1 receptor in human OA chondrocytes and synovial fibroblasts promotes binding of IL-1β (Shlopov et al., 2000). Therefore, the effects of mediators such as pro-inflammatory molecules produced by IL-1β are enhanced, resulting in joint pain, inflammation, and cartilage destruction. In animal models, the severity of OA is effectively alleviated by inhibiting IL-1β (Kloppenburg et al., 2019). Interferons are a family of naturally secreted proteins with immunomodulatory functions (van Holten et al., 2005). Upregulation of TLR2 and TLR4 is related to IFN-γ stimulation, with the involvement of a pro-inflammatory cytokine member of the IL-1 family (Radstake et al., 2004). TNF-α is one of the most important signaling molecules in the TNF family. It can be united by a specific TNF receptor (TNFR I or p55) on the cell membrane and is involved in driving the inflammatory cascade. The expression of TNFRI increased significantly in OA chondrocytes and synovial fibroblasts (Hosseinzadeh et al., 2016). Both TNF and IL-1 stimulate the chondrocytes to release cartilage hydrolases, such as matrix metalloproteinase, which is a key regulatory factor for cartilage destruction, thus promoting OA. IL-6 is a multi-functional cytokine with biological activity in immune regulation and inflammation mediation. In normal chondrocytes, it is secreted at low levels. Nevertheless, it is highly expressed in the synovial fluid of OA patients (Blasioli and Kaplan, 2014). At the same time, the secretion of IL-6 is stimulated and increased by high levels of IL-1β (Kapoor et al., 2011).

Using *in vivo* experiments, we found that the expressions of IL-1β, IL-6,TNF-α, and IFN-γ in the blood and articular cartilage of the MC group were higher than those in the BC and ZFTG groups. In the *in vitro* experiments, the supernatant of the chondrocytes in the IL-1β group also detected high expression of IL-1β, IL-6, and TNF-α. In addition, after ZFTG intervention, the expression levels of inflammatory factors reduced significantly, similar to the results of the *in vivo* experiments. This not only verifies that various factors are involved in the formation of OA but also demonstrates that ZFTG has an anti-inflammatory effect. In the ZFTG groups, the concentration gradient was negatively correlated with the expression of each factor, which also provided a reference for our subsequent *in vitro* experiments.

IL-1, IL-6, TNF, and other cytokines are encoded and expressed by the NF-κB classical pathway (Karin and Greten, 2005), which is based on IKKβ-dependent IκB degradation. Furthermore, the NF-κB family can be activated by pro-inflammatory cytokines, such as TNF-a, IL-1, and PAMPs, to coordinate a wide range of stress-like inflammatory responses (Bonizzi and Karin, 2004). In mammals, the NF-κB family consists of five distinct members (RelA (p65), RelB, C-Rel, NF κ B1 (P105/P50), and NF κ B2 (P100/P52)) (Bonizzi and Karin, 2004). The expression of related genes triggered by non-coding RNA (NC RNAs) can widely activate the NF-κB signaling pathway and induce joint damage, leading to occurrence and development of OA (Feng and Wu, 2022). There is increasing evidence that the NF-κB family plays a central role in the pro-inflammatory stress response of chondrocytes and controls their differentiation processes (Marcu et al., 2010). Many studies have effectively reduced chondrocyte apoptosis and alleviated the symptoms of OA by inhibiting the NF-κB signaling pathway, making significant progress in researching potential therapeutic drugs for OA (Zhu et al., 2021; Chen et al., 2022; Jia et al., 2022; Ni et al., 2022; Shi et al., 2022).

As an upstream target of NF-κ B and an important component of the innate immune mechanism, TLRs also play a crucial role in OA. With the discovery of the anti-inflammatory effects of TLR10 (Tang et al., 2018), the role of TLRs in OA research has become clearer. As a pattern recognition receptor, TLRs can be activated by pathogen-associated molecular patterns (PAMP) and damage-associated molecular patterns (DAMP) (Xie et al., 2019). Sensitized TLRs and some cytokines such as TNF-α can activate synovial cells and further promote inflammatory damage in the joint (Geurts et al., 2011). Various cell fragments and contents caused by inflammatory injury become new DAMPs, recognized by TLRs until the macrophages are activated to eliminate stimulation and promote tissue repair (Spite et al., 2014). TLR2 and TLR4 are the most thoroughly studied TLRs. Among them, TLR4, the only transmembrane in the family, is located on the human chromosome 9q32-q33 and is also the first mammalian TLR identified in human proteins (Keshava Prasad et al., 2009). TLR4 mostly exists as a homodimer, whereas TLR2 mostly exists as a heterodimer composed of TLR1, TLR6, and possibly TLR10. Both TLR2 and TLR4 signaling pathways can be activated by DAMPs (Elshabrawy et al., 2017). TLR4 dimers induced by ligands can activate two important signaling pathways, the MyD88-dependent and MyD88-independent, wherein the MyD88-dependent pathway is a common channel for TLRs, mainly because MyD88 was assembled to aggregate and interact with IL-1 receptor-associated kinase (IRAKs) to activate downstream NF-κB signaling and promote innate immune response (Gómez et al., 2015). Studies on the NF-κB signaling pathway usually focus on observing the nuclear translocation of NF-κB p65, which is the most prevalent and functional member of the NF-κB family. The most prevalent NF-κB inhibitory protein, IκB-α, binds to p65, causing it to reside in an inactive state outside the nucleus. Under the influence of stimulation factors, p65 and IκB-α get separated, and the former is moved into the nucleus, ultimately leading to chondrocyte degeneration and apoptosis and degradation of the matrix of the articular cartilage (Choi et al., 2019). *In vivo* experiments found that the TLR4/MyD88/NF-κB signaling pathway was activated in the model group, similar to previous studies (Li Z et al., 2020). The protein expression and gene detection results of TLR4, MyD88, and NF-κB in the ZFTG groups indicated that ZFTG did inhibit the TLR4/MyD88/NF-κB signaling pathway. This is also confirmed by the intranuclear expression of NF-κB p65. In a similar study (Ma et al., 2021), the inhibition of the TLR-4/NF-κB pathway effectively reduced cartilage degradation and inflammatory response in rats.

From our *in vitro* experiments, the high expressions of genes and proteins of TLR2, TLR4, and MyD88 in the IL-1β group were consistent with the results of Liu et al. (2017). The intervention of ZFTG significantly inhibited the expression of TLR2, TLR4, and MyD88. At the same time, considering the effects of ZFTG on chondrocytes under the same conditions, ZFTG did regulate the inflammatory damage of articular chondrocytes and alleviated apoptosis by inhibiting the TLR2-4/MyD88/NF-κB signaling pathway, consistent with other *in vitro* studies (Liu L et al., 2021) that demonstrated that the TLR4/MyD88/NF-κB signaling pathway is the inflammation signaling pathway for treatment of OA. Moreover, the TLR2/TLR4/MyD88 gene was involved in chondrocyte differentiation and metabolism by studying the TLR-2/TLR-4 double knockout and MyD88 knockout mice (Liu-Bryan and Terkeltaub, 2010). Korotkyi et al. (2021) detected the increased expression of TLR2, TLR4, and NF-κB1 genes in experimental OA rats. Another *in vitro* study demonstrated that the aggrecan 32-mer fragment induces the TLR-2-dependent gene to activate NF-κB in mouse and human chondrocytes, accelerating cartilage destruction (Lees et al., 2015). Altogether, the results of this study confirm the abovementioned studies.

It is worth noting the limitations of this study. First, ZFTG is produced by a Chinese patent medicine that uses modern equipment. However, the complexity of Chinese medicinal components makes it difficult to explore their effective mechanisms. Second, this study focused on the anti-inflammatory effect of ZFTG on KOA without comprehensively exploring the efficacy mechanism. Finally, further in-depth research is warranted to clarify the efficacy of ZFTG in treating KOA.

## 5 Conclusion

The modified Videman method provided the experimental model of OA for this study. ZFTG inhibits IL-1β-induced inflammatory injury in rabbit chondrocytes by repressing the TLR4/MyD88/NF-κB signaling pathway, secreting inflammatory factors, and promoting the survival of chondrocytes while reducing the apoptosis of chondrocytes. In summary, TCM is a potential reservoir for prevention and treatment of KOA. Our study not only provides an important reference for treatment of KOA, which reveals that ZFTG can be used as a new drug and its curative mechanism, but also clarifies the vital function of the TLR4/MyD88/NF-κB signaling pathway in KOA. Our study can provide the direction for modern Chinese medicine research, provide the basis for the clinical treatment of KOA, and lay the foundation for the TCM treatment of KOA.

## Data Availability

The original contributions presented in the study are included in the article/Supplementary Materials; further inquiries can be directed to the corresponding authors.
